# Shifting in the global flood timing

**DOI:** 10.1038/s41598-022-23748-y

**Published:** 2022-11-07

**Authors:** Gonghuan Fang, Jing Yang, Zhi Li, Yaning Chen, Weili Duan, Charles Amory, Philippe De Maeyer

**Affiliations:** 1 State Key Laboratory of Desert and Oasis Ecology, Xinjiang Institute of Ecology and Geography, Chinese Academy of Sciences, 818 South Beijing Road, Urumqi, 830011 China; 2 Sino-Belgian Joint Laboratory for Geo-Information, Urumqi, 830011 China; 3 Xinjiang Key Laboratory of Water Cycle and Utilization in Arid Zone, Urumqi, China; 4 National Institute of Water and Atmospheric Research, Christchurch, 8000 New Zealand; 5 CNRS, Institut des Géosciences de l’Environnement, University Grenoble Alpes, 38100 Grenoble, France; 6 Department of Geography, Ghent University, 9000 Ghent, Belgium; 7 Sino-Belgian Joint Laboratory for Geo-Information, 9000 Ghent, Belgium

**Keywords:** Hydrology, Natural hazards, Climate-change impacts

## Abstract

Climate change will have an impact on not only flood magnitude but also on flood timing. This paper studies the shifting in flood timing at 6167 gauging stations from 1970 to 2010, globally. The shift in flood timing and its relationship with three influential factors (maximum 7-day precipitation, soil moisture excess, and snowmelt) are investigated. There is a clear global pattern in the mean flooding date: winter (Dec–Feb) across the western Coastal America, western Europe and the Mediterranean region, summer (Jun–Aug) in the north America, the Alps, Indian Peninsula, central Asia, Japan, and austral summer (Dec–Feb) in south Africa and north Australia area. The shift in flood timing has a trend from − 22 days per decade (earlier) to 28 days per decade (delayed). Earlier floods were found extensively in the north America, Europe and northeast Australia while delayed floods were prevailing in the Amazon, Cerrado, south Africa, India and Japan. Earlier flood timing in the north America and Europe was caused by earlier snowmelt while delayed extreme soil moisture excess and precipitation have jointly led to delayed floods around the monsoon zone, including south Africa, India and Japan. This study provides an insight on the shifting mechanism of flood timing, and supports decisions on the global flood mitigation and the impact from future climate change.

## Introduction

Floods are one of the most dangerous climate-related disasters, and climate change has altered the distribution, intensity and timing of floods worldwide^1–7^.

There are intensive studies on flooding, most of which focus on the historical trends in flood magnitude and intensity and could not reach a consistent signal in flood magnitude change on global scale^8–14^. The influencing factors in flood magnitude changes have also been investigated extensively including climatic and human factors, e.g., precipitation, soil moisture, snow, dam construction, land-use change, river training^14–18^. Recently, there have been more studies on flood timing^3,19–25^. The spatial pattern of mean flooding date has been identified in Europe^3,19,22,26^, north America^23,27–29^, Australia^30^, Brazil^31^, Africa^32^ or globally^10,21^.

Shifts in flood timing is often used as a monitor to interpret the mechanisms that cause floods^20,33^. The underlying mechanism under exploitation includes the atmospheric conditions and others, and the atmospheric factors are much more influential than the catchment and river system factors^11,23,34^. For a specific flood event, the influential factors are well documented in terms of hydro-meteorological or circulation aspect^35,36^. On the regional or continent scales, these influential factors include short-term heavy rainfall, rainfall on saturated soil, snow melt water, which individually or jointly stimulate the flood events^3,37–39^. For example, the mean flood date has altered especially in the high latitude north hemisphere due to warming induced earlier snowmelt^3,19^. In Europe, earlier spring snowmelt floods in northeastern Europe, delayed winter flood around the North Sea and parts of the Mediterranean coast and earlier winter flood in Western Europe were abserved and most annual floods are caused by subextreme precipitation with high antecedent soil moisture, not by annual peak rainfall^3,19^. In the Upper Austria, one- or seven-day extreme precipitation is usually a better covariate for variations of the flood frequency curve than precipitation on longer time scales^34^. In the United States, the flood mechanisms can be summarized as daily precipitation, weekly precipitation, precipitation excess and snowmelt plus rainfall^20^. In Australia, changes in antecedent soil moisture modulate flood seasonality and the driving factors are different in small and large floods^30^. Some studies also attribute flood time change to large scale circulations^12,40^. Most of these studies focus on changes in flood timing on a continental or basin scales, the research on the spatial pattern of shifts in flood timing under different climatic conditions on the global perspective is still unsufficient^30,33^.

The objective of this study is to quantify the shifts in flood timing during the past few decades and to identify its influencing factors at a global scale considering the climatic zones. Based on the map of flood timing changes, we quasi-quantitatively analyze the occurrence time of three potential influential factors, i.e. the extreme precipitation, soil moisture excess and snowmelt and infer the main causes leading to the flood timing shifts.

## Results

### Spatial distribution of mean flooding time and its concentration index

**Figure 1:**
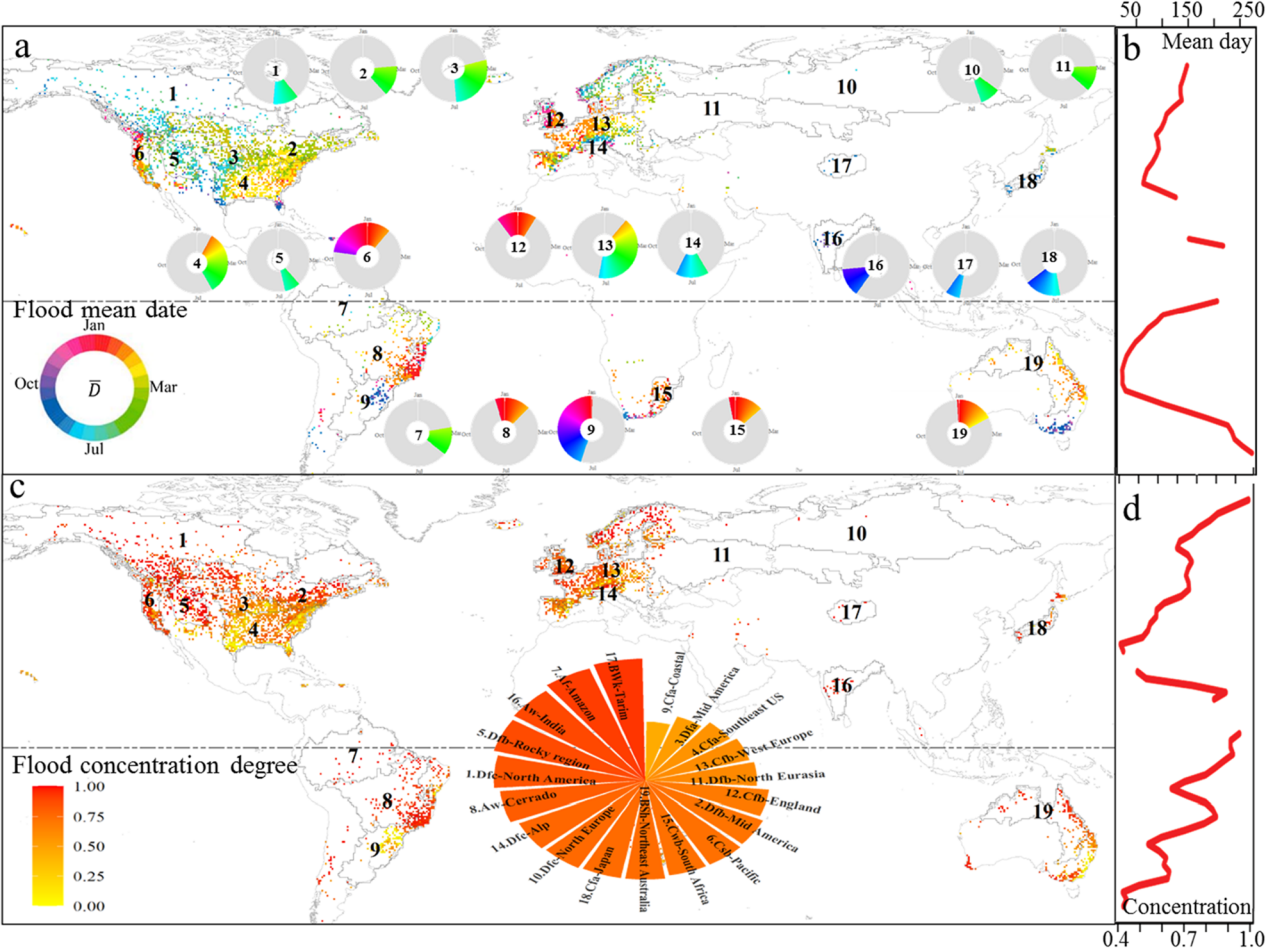
The spatial pattern of flood timing during 1970–2010: (**a**) mean flood date, displayed as circular for each hotspot; (**b**) latitude averaged mean flood date; (**c**) flood concentration index around corresponding mean occurrence date; (**d**) latitude averaged flood concentration. Both the mean and concentration of flooding date are aggregated in 19 hotspots based on the Köeppen Climate Classification System^43^. The names of these hotspots are listed below: 1. Dfc-North America, 2. Dfb-Mid America, 3. Dfa-Mid America, 4. Cfa-Southeast US, 5. Dfb-Rocky region, 6. Csb-Pacific,7. Am-Amazon, 8. Aw-Cerrado, 9. Cfa-Coastal, 10. Dfc-North Europe, 11. Dfb-North Eurasia, 12. Cfb-England, 13. Cfb-West Europe, 14. Dfc-Alp, 15. Cwb-South Africa, 16. Aw-India, 17. BWk-Tarim, 18. Cfa-Japan, 19. BSh-Northeast Australia) (Am/Aw is equatorial monsoon/winter dry climate, BWk/BSh is arid desert cold/hot climate, Cfa/Cfb and Csb/Cwb is warm temperate fully humid hot/warm summer and summer/winter dry warm summer climate, Dfa/Dfb/Dfc is snow fully humid hot/warm/cool summer climate).

There is a distinct regional pattern in the spatial distribution of the mean date of occurrence and concentration index of global floods pattern (Fig. 1). In the Northern Hemisphere, there is a clear transition of flood timing in the latitudinal direction except for some regions suffering from regional topographical or monsoon effects (Fig. 1b). Winter flooding typically takes place across the western coast of America, large parts of Western Europe and the Mediterranean (with high concentration), while spring flooding generally takes place in eastern and southeastern America and western continental Europe. It is worth noting that the concentration index of floods in southeast America is quite low, meaning that flood timing in this region is widely dispersed throughout the year due to a combination of tropical and extratropical systems controlling the flooding^23^. Summer flooding is found around north America, the Alps, India and Japan. These patterns have been described in detail in regional studies^3,20,22,41^. In southern Hemisphere, the available flood timing data were mainly available in South Africa, Australia and Brazil. Flooding normally occurs in December, January and February in south Africa, north Australian and central Brazil (with concentration index approaching 1.0 in these regions in Fig. 1c). An interesting phenomenon is observed in Brazil: a contrasting flood behavior is observed in the adjacent Cerrado and Coastal regions, as flood in the coastal regions was jointly influenced by cold fronts, thunderstorms, and tropical cyclones, which make rainfall-induced floods occurring throughout the year, which leads to a high variability in flood timing with a low concentration (R = 0.31)^25,31,42^.

Though the global flood timing exhibits strong seasonality at most stations (i.e., 4590 stations, out of a total of 6167 stations, have a concentration index larger than 0.5 and 2764 stations 0.7), low concentration (i.e., without a specific flooding season) was observed in several hotspots, for example, Hotspot 3 and 4 (mid and southeast United States), Hotspot 9 (Costal of South America), and certain hotspots in Europe (11. Dfb-North Eurasia, 12. Cfb-England and 13. Cfb-West Europe) due to the combined system as mentioned above^22,23^ (Fig. 1c). Stations at high latitude north hemisphere and equatorial areas showed high concentration while stations at high latitude south hemisphere demonstrated low concentration (Fig. 1d). We excluded these hotspots with concentration indexes less than 0.7 when studying the influential factors in flood timing shifts.

### Shift in flood timing

**Figure 2:**
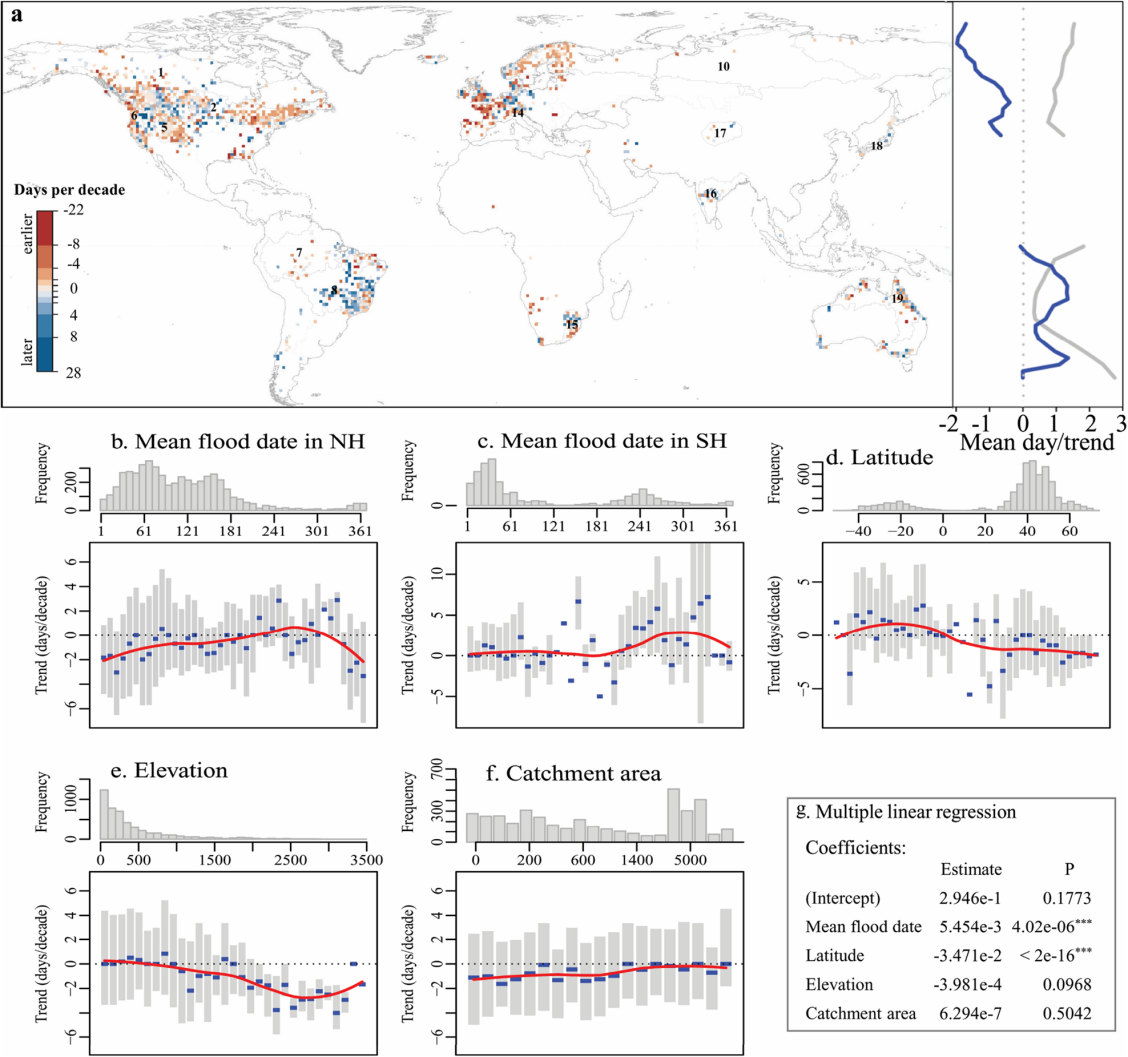
Global distribution of the trend in observed flood timing during 1970–2010 with latitude average flooding date (gray line; for display purposes the magnitude of the flood date is scaled by a factor of 0.01) and trend in flood timing (blue line) (**a**). Boxplots *b-f* demonstrate the relations between the trend in flood timing and mean flood date in the northern hemisphere (NH) (**b**) and southern hemisphere (SH) (**c**), latitude (**d**), outlet elevation (**e**) and catchment area (**f**). The interquartile range, equaling the difference between the 25th and 75th percentiles, is used to characterize variability for each bin. The red lines represent a loess-curve fitted to the values. The station density was also displayed in subplot (**b**–**f**). Subplot (**g**) shows the multiple linear regression analysis between flood timing trend with flood mean date, latitude, elevation and area.

Figure 2A shows the spatial patterns in the changes of global flood timing during 1970–2010. The trend in flood timing ranges from − 22 days per decade towards earlier floods to 28 days per decade towards delayed floods using the adjusted Theil-Sens slope. Floods tend to occur earlier in north and mid-America, the Mexican Gulf region, Great Lake, north of Australia and large parts of Europe. In contrary, at the Amazon, southern Brazil, India, Japan and northest Austrialia, flood timing tends to be delayed (Table S1).

To some extent, the trend in flood timing is significantly related to mean date of floods and catchment latitude (Fig. 2b, c, d, g). Generally, Winter/spring flooding (approx. day 330 ~ 61 in the northern hemisphere) tends to occur earlier (Fig. 2b, c) (e.g. Hotspots 1, 2, 5, 6, 10, 11, 12, 13), while a summer or early autumn flooding (e.g. Spots 16, 18) is normally featured with delayed flood peaks. The higher the latitude, the earlier the flood timing in the north hemisphere (Fig. 2d). Catchment elevation and area also have an impact on flood timing but their effects were not statistically significant (Fig. 2e, f, g).

### Influencing factors on the shifting of flood timing

**Figure 3:**
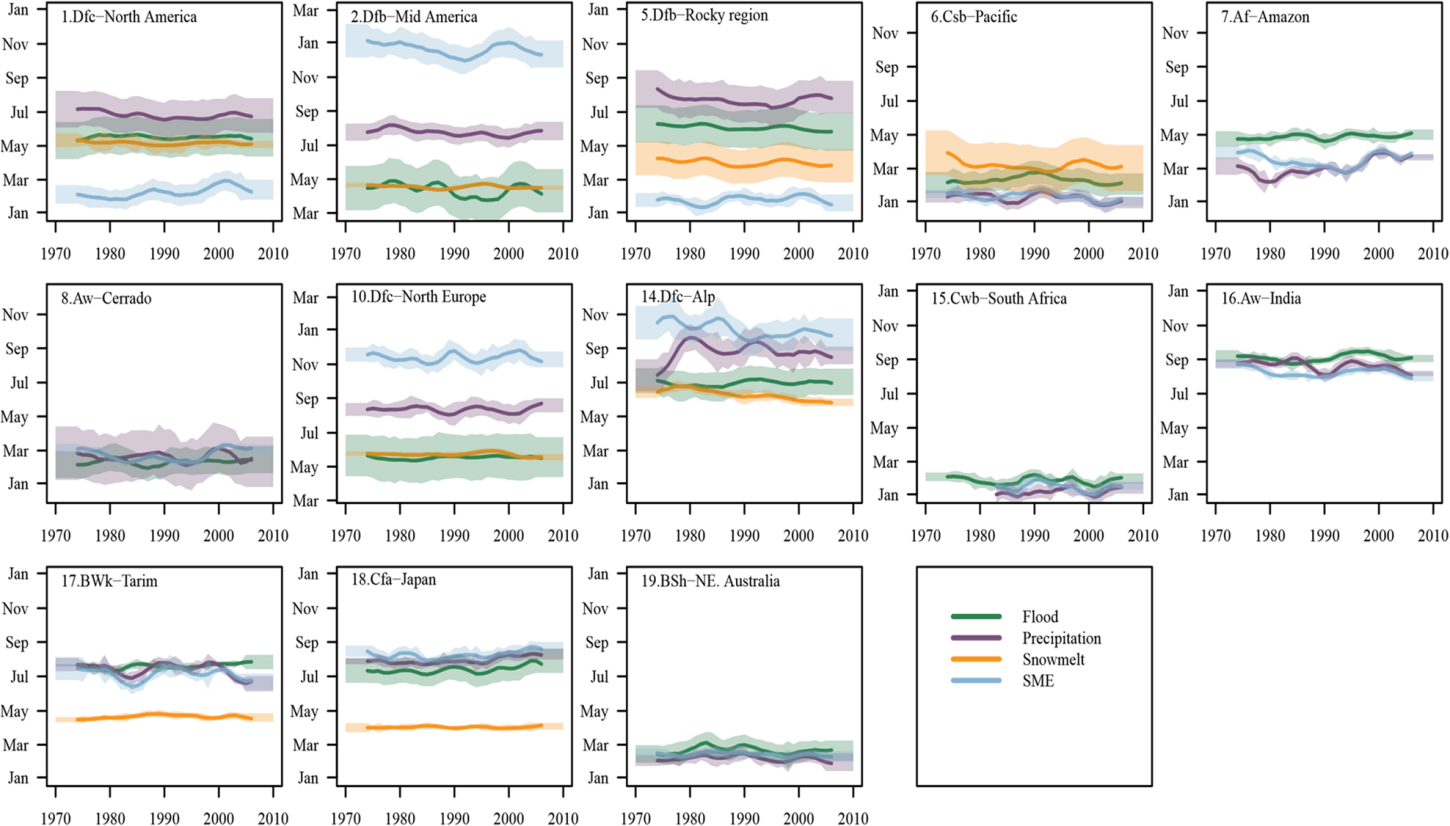
Long-term temporal evolutions of flood timings and their influential factors for 13 hotspots with concentrate index > 0.7. Solid lines show the median timing over the entire hotspot: Green: timing of the observed floods; purple: 7-day maximum precipitation; orange: snow melting indicator and blue: timing of the calculated maximum soil moisture excess (SME). All data were subjected to a 10-year weighted moving average filter. The shaded bands indicate the timing variability within the year (± 0.5 standard deviations). Vertical axes show month of the year (note different starting months in these panels). The precipitation and SME data were started from 1979 in south africa as the CPC precipitation data was started from 1979.

As floods are the results of the interplay between the precipitation, soil moisture excess and snow processes^3,44^, below we analyzed the shifting in these variables and their relations with flood timings. For the north and mid-America and north Europe (Hotspot 1, 2, 10), the spring snowmelt generally occurs during spring or early summer (Fig. 3). In these regions, the temporal changes of the timing of snowmelt maxima matches well with the timing of the floods, which lines up with previous regional studies^22,45^. At the west Pacific coast of America (Hotspot 6), Amazon (Hotspot 7), Cerrado Savanna (Hotspot 8), South Africa (Hotspot 15), India (Hotspot 16) and Northeast Australia (Hotspot 19), the precipitation and soil moisture excess could occur during the same period in a year (within 10 days lag), and the extreme precipitation and soil moisture excess (normally driven by a several-day heavy precipitation) jointly contributed to the floods, which could be inferred from the similar changing pattern and contemporaneous occurrence times of the extreme precipitation, soil moisture excess and floods (Fig. 3). Coincidentally, these hotspots with delayed flood timing are located in the monsoon dominated hotspots, including south Africa (Hotspot 15), India (Hotspot 16), Japan (Hotspot 18) and the Amazon areas (Hotspot 7).

## Discussion

This study explored the spatial pattern of global flood timing, its shifting and reasons. Compared to those studies on the mean flood date and flood-timing shifting in Europe and north America, this study lines up with the existing literature^3,23,24,46^.

On a global or regional scale, one could analyze the causes behind the observed pattern based on the overlapped time window of floods and other factors. For example, the earlier floods in Scandinavia (in Hotspot 10) are likely to be predominately influenced by snowmelt, of which the timing of the maximum 7-day value is the same with the flood timing (Fig. 3). This method has proven to be effective and relatively simple and practical compared to hydrological modelling or earth-system simulations in interpreting the flood mechanisms^3,20^.

The flood timing is expected to occur later in the monsoon dominated regions despite few sites showing statistically significant trends (Figure S1a). For South Africa, the spatial pattern of the flood timing changes (based on limited stations) is similar with those based on the reanalysis data^32^. For India and Japan, the flood is more related to the monsoon indicators and delayed flood peaks have been observed, of which may be related to increased atmospheric effective specific capacity and slower response to seasonal solar forcing^47^. For the south of Brazil (HS 8, Cerrado), the flood timing is getting delayed, which is consistent with the analyzed outcomes based on the local water-year flood^10^. In future studies in the monsoon regions, more focus should be on the relation between the floods and large-scale circulation conditions as floods may be more dependent on these circulations^32^.

The flood timing occurred earlier in the snow melting dominated regions (Figure S1b), such as the Great Lake region (HS 2), Scandinavian region (HS 10), East Germany (HS 13), South English Channel and the United Kingdom region (HS12). The continuous global warming is considered a possible mechanism pushing forward the snow melting floods^46,48^. If the snow processes become less important in the future, the flood seasonality patterns in these regions may alter completely as other flood generation processes become dominant, e.g., more rain on snow events^22^.

The changes in soil moisture excess have also resulted in earlier floods in some tropical or warm temperate areas, such as North Australia (HS 19). Soil moisture excess should be a more realistic factor to predict floods compared to the precipitation in several regions (e.g., South Africa[HS15], northest Australia [HS19]), as the timing of soil moisture excess coincide better with flood timing.

Despite above promising result, there is still some space for future improvement. For example, only the timing of the annual 7-day maximum flood was studied, which excludes the fact that there might be more than one flood peak for some stations (e.g. two flood seasons). The regional interpretation of flood timing shifts is influenced by the limited and non-uniform spatial coverage, inconsistency of datasets and number of hydrometric stations, which causes high uncertainty, especially for the data-scarce region. Additionally, the flood timing in each calendar year rather than water year is recorded in GISM, which also increased the uncertainty in certain stations with a sequence of dependent flood events being counted in two successive years. Nested catchments were not identified. Furthermore, the combined effect from rainfall and soil moisture excess has not been distinguished yet.

Flood timing shifting will certainly have implications on water management practice and food security. For regions with a strong signal of flood timing changes, local practices of flood management should be adjusted to adapt to the shifted flood peaks, such as preparing for the earlier flood period, discharging reservoirs for later flood^49^. Another practical influence of shifted flood timing is closely related to food security. The water shortage may be prevailing during the water demanding season due to earlier or later flood peaks, resulting in reduction of crop yield and becoming a threat to food security.

## Conclusions

This work provides a study on global flood timing shifts and and contributions from three main influential factors (i.e., maximum 7-day precipitation, soil moisture excess, and snowmelt). The shifts in flood timing are calculated with circular index based on the observed flood data from 1970 to 2010. The causality relationship of flood timing and its three influential factors were investigated based on visual interpretation.

Despite the difference in the spatial distribution of the flood timing shifts, there are some spatial patterns: Earlier floods are more prevailing in high-latitude regions, mainly caused by earlier snowmelt due to global warming. Regions with delayed floods were mainly distributed in the monsoon-affected areas (e.g., South Africa, India and Japan), where the delayed floods were mainly the interplay of delayed rainstorm and soil moisture excess.

Future climate change will have a big impact on the global precipitation and snowmelt. This will eventually lead to the shifting on global flood time. This study provides an insight on the shifting mechanism of flood timing, and could be used to supports decisions on the global flood mitigation and the impact from future climate change.

## Data and methods

### Data

In this study, we analyzed a large data set of flood observations to study the shifting in flood timing at 6167 gauging stations from 1970 to 2010. The Global Streamflow Indices and Metadata (GSIM) archive and the Global Runoff Data Centre (GRDC) dataset were jointly used.

For climatic variables, daily precipitation data from the Global Historical Climatology Network (GHCN), the Asian Precipitation Highly Resolved Observational Data Integration Towards Evaluation of Water Resources (APHRODITE), the CPC Global Unified Precipitation data provided by the NOAA/OAR/ESRL PSL, South America Daily Gridded Precipitation (1979 ~) and the NCEP/NCAR Reanalysis Products 1 were also used. The CRU TS4.05 maximum and minimum temperature dataset was interpolated to daily scale using spline interpolation method in R Package “stats”. The daily temperature data were used to calculate the potential evapotranspiration and snowmelt.

We restricted our analysis to sites with no less than 37 years of data (flood) during period 1970–2010 (allowing for a maximum of 4 years’ missing data), resulting in a total of 6167 sites across the world. The study period is selected based on data availability. For Africa and Asia, few gauges were used due to data availability. Note that for the south America, the CPC precipitation only records daily precipitation during 1979 to recent. More details on the datasets and preprocessing are described in Text S1 in Supporting Information.

### Definition of flood and its influential factors

In this study, floods are represented by the annual maximum 7-day flow. Regarding the flood timing influential factors, we assume floods were only caused by the following climatic factors, i.e. the precipitation, soil moisture excess and snow melt, since most floods are caused by strong rainfall, strong snowmelt or rain on saturated soil^3,19,20,44^. Only natural process factors were being analyzed, omitting human factors such as land use change and engineering of rivers, which are however indirectly reflected in the gauged data.

*Extreme precipitation* in this case, we assume that floods were resulted from the largest 7-day precipitation. Moreover, the occurring times of flood and extreme precipitation should fall into the same period of the year.

*Soil moisture excess* in this case, floods are triggered by high antecedent soil moisture conditions and a strong rainfall during long periods (up to months) rather than short duration rainfall events. The soil moisture excess combines daily precipitation, evapotranspiration, and the antecedent soil moisture and is defined as the daily precipitation amount minus the available soil moisture storage capacity^16,19,48^. Soil moisture excess can also be referred to as surface runoff based on the calculation procedures (Eqs. 1, 2).

1 $$Su\left( t \right) = {\text{max}}\left[ {{\text{min}}\left( {Su\left( {t - 1} \right) + P\left( t \right), Su_{max} } \right) - E\left( t \right),0} \right]$$

2 $$E\left( t \right) = {\text{min}}\left[ {0.75 \times ET0,{\text{ Su}}\left( {t - 1} \right)} \right]$$

where *Su*(*t*) and *Su*(*t* − 1) represent the soil moisture storages on day *t* and day *t* − 1. $Su_{max}$ is the maximum soil moisture storage fixed at 25 mm. Changing this to 75 mm led to the soil moisture excess being 0 for most days as the precipitation will always smaller than evaporation. The changes of the maximum soil moisture did not substantially affect the global results^19^. P(*t*) and E(*t*) demonstrate the precipitation and evapotranspiration amount on day *t*, respectively*. ET0* is the reference evapotranspiration, calculated by the Hargreaves-Samani method in the R *evapotranspiration* package^50,51^.* E* is scaled to 75% of its daily value because not all ET0 were utilized for evapotranspiration.

*Snowmelt* In snowmelt induced floods, the maximum annual flow is assumed to be caused by the largest 7-day snowmelt. The latter is calculated based on an empirical degree-day model^51^.

3 $$Ss\left( t \right){ } = \left\{ {\begin{array}{*{20}l} {min\left( {fdd*\left( {TT\left( t \right) - t_{crit} } \right),snowpack\left( {t - 1} \right)} \right)} \hfill & {TT\left( t \right) > t_{crit} } \hfill \\ 0 \hfill & {TT\left( t \right) \le t_{crit} } \hfill \\ \end{array} } \right.$$

4 $$snowpack\left( t \right) = \left\{ {\begin{array}{*{20}c} {snowpack\left( {t - 1} \right) - Ss\left( t \right),} & {TT\left( t \right) > t_{crit} } \\ {snowpack\left( {t - 1} \right) + P\left( t \right),} & {TT\left( t \right) \le t_{crit} } \\ \end{array} } \right.$$

where *Ss*(*t*) and $snowpack\left( t \right)$ are the snowmelt and snow storage on day *t* (mm), respectively. $TT\left( t \right)$ is the daily maximum temperature on day *t*, $t_{crit}$ the temperature threshold for snowmelt and for the delimiting rainfall and snowfall, which is set at 5 °C. *fdd* is the snow melting factor, established at 2 mm per day per °C^19^.

### Seasonality characteristics of the flood date and its influencing factors

In this study, we calculated the mean flooding date and the flood concentration at each station. Then the trend in the flooding timing is estimated using the adjusted Theil-Sen slope method.

The seasonality of flooding and its influencing factors is characterized by circular statistics^3,19,20,24^. When applying this analysis, we firstly excluded the flow stations whose floods are uniformly distributed within the year (through the Kuiper’s test with a significance level 0.05) and 5763 stations were used in the following analysis. To calculate the mean flood date and its concentration statistics, the flood occurrence date, *D*_*i*_ (day of the year), was converted into an angular value $\theta_{i}$. For station *i,*

5 $$\theta_{i} = D_{i } \cdot \frac{2\pi }{{m_{i} }}$$

where $D_{i }$ = 1 corresponds to January 1st and $D_{i }$ = $m_{i}$ to December 31st, and $m_{i}$ is the Julian days in year *i (i.e.* 365 in regular years and 366 in leap years). The average date of occurrence $\overline{D}$ of flood (at a station) is defined as:

6 $$\overline{D} = \left\{ {\begin{array}{*{20}l} {tan^{ - 1} \left( {\frac{{\overline{y}}}{{\overline{x}}}} \right) \cdot \frac{{\overline{m}}}{2\pi },} \hfill & {\overline{x} > 0,\overline{y} > 0} \hfill \\ {\left[ {tan^{ - 1} \left( {\frac{{\overline{y}}}{{\overline{x}}}} \right) + \pi } \right] \cdot \frac{{\overline{m}}}{2\pi },} \hfill & {\overline{x} \le 0} \hfill \\ {\left[ {tan^{ - 1} \left( {\frac{{\overline{y}}}{{\overline{x}}}} \right) + 2\pi } \right] \cdot \frac{{\overline{m}}}{2\pi },} \hfill & {\overline{x} > 0,\overline{y} < 0} \hfill \\ \end{array} } \right.$$

where $\overline{x} = \frac{1}{n}\sum\nolimits_{i = 1}^{n} {cos\left( {\theta_{i} } \right)}$ and $\overline{y} = \frac{1}{n}\sum\nolimits_{i = 1}^{n} s in\left( {\theta_{i} } \right)$ ($\theta_{i}$ is defined in Eq. 5) are the cosine and sine components of the average date, respectively, $\overline{m} = \frac{1}{n}\sum\nolimits_{i = 1}^{n} {m_{i} }$ represents the average number of days per year and *n* the total number of floods at each station^3^.

To illustrate the variability of the flood occurrence date, a concentration statistic R of the occurrence date around the average date ($\overline{D}$) is defined:

7 $$R = \sqrt {\overline{x}^{2} + \overline{y}^{2} } ,\quad 0 \le R \le 1$$

where *R* = 0 indicates that flood occurrence dates are widely dispersed or sitting opposite on the unit circle throughout the year, and *R* = 1 indicates that all flooding events are concentrated on the same ordinal day.

To quantify the trend in the timing of floods and meteorological divers, the adjusted Theil-Sen slope estimator was applied^52^. This non-parametric estimator *β* was chosen for its robustness and insensitivity to missing values and outliers. The trend estimator *β* was estimated as:

8 $$\beta = median\left( {\frac{{D_{j} - D_{i} + k}}{j - i}} \right)$$

where $$k = \left\{ {\begin{array}{*{20}l} { - \overline{m},} \hfill & {D_{j} - D_{i} > \frac{{\overline{m}}}{2}} \hfill \\ {\overline{m}, } \hfill & {D_{j} - D_{i} < - \frac{{\overline{m}}}{2}} \hfill \\ {0,} \hfill & { otherwise} \hfill \\ \end{array} } \right.$$, is the adjustment factor and *β* denotes the slope of the flood timing changes^3^.

### Dominant climatic factors in flood timing changes

In this study, 19 hotspots are identified according to the Köeppen–Geiger classification^43^ and the similarity of mean flood dates (Fig. 1). Names of these hotspots consist of the climate zone and geographical location. Note that the names are only indicative for a region and do not exactly correspond to any exactly defined geographic area.

Since the available flood data are limited to the flood dates of each year instead of daily streamflow, we identified the dominant flood-influencing factor by comparing the flood dates with the timing of each candidate mechanism^19^. Our assumption is that two variables are causally related if they are in the same occurrence time window (i.e., occur simultaneously) and have similar trends.

## Supplementary Information

Supplementary Information.

## Data Availability

The flood date data used in this study can be downloaded from https://doi.pangaea.de/10.1594/PANGAEA.887477 and https://www.bafg.de/GRDC/EN/Home/homepage_node.html. The precipitation can be downloaded from https://www.ncei.noaa.gov/products/land-based-station/global-historical-climatology-network-daily for America, Europe and Australia, from http://aphrodite.st.hirosaki-u.ac.jp/products.html for Asia region, from https://psl.noaa.gov/data/gridded/data.cpc.globalprecip.html and https://psl.noaa.gov/data/gridded/data.ncep.reanalysis.html for south America. The temperature data can be downloaded from https://crudata.uea.ac.uk/cru/data/hrg/cru_ts_4.05/.
