# Supplementary material for: Shifting in the global flood timing
Source: Sci Rep. 2022 Nov 7;12:18853. doi: 10.1038/s41598-022-23748-y (PMC9640645; doi:10.1038/s41598-022-23748-y)
Supplement: Supplementary file 1 — Supplementary Information. [file 41598_2022_23748_MOESM1_ESM.docx]

Supporting Information for

**Shifting in the Global Flood Timing**

G. Fang^1,5^, Z. Li^1*^, J Yang^2^, Y. Chen^1^, W. Duan^1^, C. Amory^3^, P. De Maeyer^4,5^

1 State Key Laboratory of Desert and Oasis Ecology, Xinjiang Institute of Ecology and Geography, Chinese Academy of Sciences, Urumqi 830011, China

2 National Institute of Water and Atmospheric Research, Christchurch 8000, New Zealand

3 University Grenoble Alpes, CNRS, Institut des Géosciences de l’Environnement, Grenoble, 38100, France

4 Department of Geography, Ghent University, Ghent 9000, Belgium

5 Sino-Belgian Joint Laboratory for Geo-Information, Urumqi 830011, China and Ghent 9000, Belgium

**Contents of this file**

Text S1

Tables S1

Figure S1

Text S1.

*Flood.* The timing and magnitude of 7-day maximum flow from Global Streamflow Indices and Metadata (GSIM) archive is used for the analysis of flood timing. The data can be freely downloaded at PANGEA data depository *https://doi.pangaea.de/10.1594/PANGAEA.887477* [^1^](#_ENREF_1)^,^[^2^](#_ENREF_2). This archive contains a large collection of metadata and streamflow indices from catchments around the globe and promotes the research for catchments with specific characteristics. In total, GSIM includes a worldwide collection of metadata and indices derived from more than 35,000 daily streamflow time series. In addition, hydrological stations in Global Runoff Data Centre (GRDC, *https://www.bafg.de/GRDC/EN/Home/homepage_node.html*) dataset were also included as a supplementary to GSIM. And six long term hydrological stations in arid central Asia were also included.

*Precipitation.* We obtained the daily precipitation data for the same time period from the Global Historical Climatology Network (GHCN, *https://www.ncei.noaa.gov/products/land-based-station/global-historical-climatology-network-daily*). The GHCN dataset provides quality-controlled precipitation observations with a global coverage. However, the densest historical station networks are located in the United States, Canada, Europe and Australia, while other regions have fewer stations. For the Asian regions (including Japan and India), the Asian Precipitation Highly Resolved Observational Data Integration Towards Evaluation of Water Resources (APHRODITE, http://aphrodite.st.hirosaki-u.ac.jp/products.html) Daily climate dataset during 1970-2007 was used as observation data. This dataset was created by interpolating the station observations into a 0.25°×0.25° grid with a quality control system for the Asian region [^3^](#_ENREF_3). For the African regions, the CPC Global Unified Precipitation data provided by the NOAA/OAR/ESRL PSL (*https://psl.noaa.gov/data/gridded/data.cpc.globalprecip.html*) during 1979-2010 were applied in order to detect the maximum precipitation time. For the South American region, the South America Daily Gridded Precipitation available from Physical Sciences Laboratory (PSL, *https://downloads.psl.noaa.gov/Datasets.other/south_america/*) was used and the NCEP/NCAR Reanalysis Products 1 (*https://psl.noaa.gov/data/gridded/data.ncep.reanalysis.html*) were also used as complementary data.

*Temperature.* The CRU TS4.05 maximum and minimum temperature dataset downloaded at *https://crudata.uea.ac.uk/cru/data/hrg/cru_ts_4.05/* [^4^](#_ENREF_4) was interpolated to daily scale using spline interpolation method in R Package “stats”. The daily temperature data were used to calculate the potential evapotranspiration and snowmelt.

Table S1. Statistics of worldwide flood timing summarized in 19 hotspots of the Köeppen Climate Classification System [^5^](#_ENREF_5). The orange-shaded and blue-shaded hotspots represent the advanced and postponed regions in flood timing, respectively. The gray-shaded rows are hotspots with concentration degree lower than 0.70.

| Hotspot Names | No. of Stations whose floods exhibit a significant seasonal distribution | Mean Flood Date (Julian Date) | Flood Concentration Degree | Sen Slope: Median [Min, Max]^a^ | No. of Stations with significant flood timing shift^b^ | Main flood generation mechanisms in literature |
| --- | --- | --- | --- | --- | --- | --- |
| 1. Dfc-North America | 253 | 166.0 | 0.85 | -0.04[-1.21,2] | 47/27 | Snowmelt and extreme precipitation [^6^](#_ENREF_6) |
| 2. Dfb-Mid America | 757 | 101.9 | 0.73 | -0.13[-2.42,2.37] | 151/96 | Snowmelt and rain-on-snow events [^7^](#_ENREF_7)^,^[^8^](#_ENREF_8) |
| 3. Dfa-Mid America | 326 | 112.8 | 0.55 | 0.27[-1.96,2.37] | 28/14 | Precipitation excess [^7^](#_ENREF_7) |
| 4. Cfa-Southeast US | 691 | 77.7 | 0.49 | -0.15[-3.38,2.9] | 68/36 | Precipitation excess [^7^](#_ENREF_7) |
| 5. Dfb-Rocky region | 263 | 154.3 | 0.88 | -0.17[-1.86,2] | 76/52 | Daily precipitation and snowmelt [^7^](#_ENREF_7) |
| 6. Csb-Pacific | 366 | 40.1 | 0.73 | -0.05[-3.02,2.34] | 34/14 | Precipitation excess [^7^](#_ENREF_7) |
| 7. Am-Amazon | 20 | 107.8 | 0.92 | 0.01[-0.62,0.4] | 0/0 | Rainfall [^9^](#_ENREF_9) |
| 8. Aw-Cerrado | 154 | 36.4 | 0.81 | 0.20[-1.1,1.84] | 26/15 | Rainfall [^9^](#_ENREF_9) |
| 9. Cfa-Coastal | 84 | 301.5 | 0.52 | 0.35[-1.07,2.63] | 12/6 | Frontal systems and convective activity [^10^](#_ENREF_10) |
| 10. Dfc-North Europe | 218 | 140.5 | 0.78 | -0.2[-2.58,1.32] | 70/45 | Snowmelt [^11^](#_ENREF_11) |
| 11. Dfb-North Eurasia | 58 | 83.4 | 0.63 | -0.45[-1.73,1.24] | 14/7 | Snowmelt [^11^](#_ENREF_11) |
| 12. Cfb-England | 170 | 11.2 | 0.69 | -0.36[-2.25,1.5] | 22/9 | Snowmelt and the concurrence of heavy precipitation with high antecedent soil moisture [^11^](#_ENREF_11) |
| 13. Cfb-West Europe | 778 | 53.9 | 0.62 | -0.05[-2.00,2.2] | 76/34 | Soil moisture excess [^11^](#_ENREF_11) |
| 14. Dfc-Alp | 90 | 180.9 | 0.77 | -0.22[-1.29,2.85] | 28/20 | Extreme precipitation [^11^](#_ENREF_11) |
| 15. Cwb-South Africa | 93 | 26.4 | 0.73 | 0.10[-1.45,1.17] | 3/1 | Indian Ocean Dipole and El Niño–Southern Oscillation [^12^](#_ENREF_12) |
| 16. Aw-India | 29 | 242.5 | 0.88 | 0.14[-0.33,0.70] | 1/0 | Severe storms and extreme precipitation [^13^](#_ENREF_13) |
| 17. BWk-Tarim | 5 | 200.3 | 0.92 | 0.05[-0.19,0.63] | 0/0 | Precipitation and glacier melt [^14^](#_ENREF_14) |
| 18. Cfa-Japan | 48 | 191.4 | 0.75 | 0.03[-0.45,0.95] | 4/0 | Short term and long term precipitation [^15^](#_ENREF_15) |
| 19. BSh-Northeast Australia | 105 | 49.9 | 0.75 | -0.11[-1.3,1.58] | 13/8 | Long term precipitation and antecedent soil moisture [^15^](#_ENREF_15)^,^[^16^](#_ENREF_16) |

### ^a^ Negative trend indicates an earlier flood timing while positive value a delayed flood timing. The median, maximum and minimum slopes are summarized within the given hotspot

^b^ No. of Stations with significant flood timing shift at significant levels of P<0.1 and P <0.05


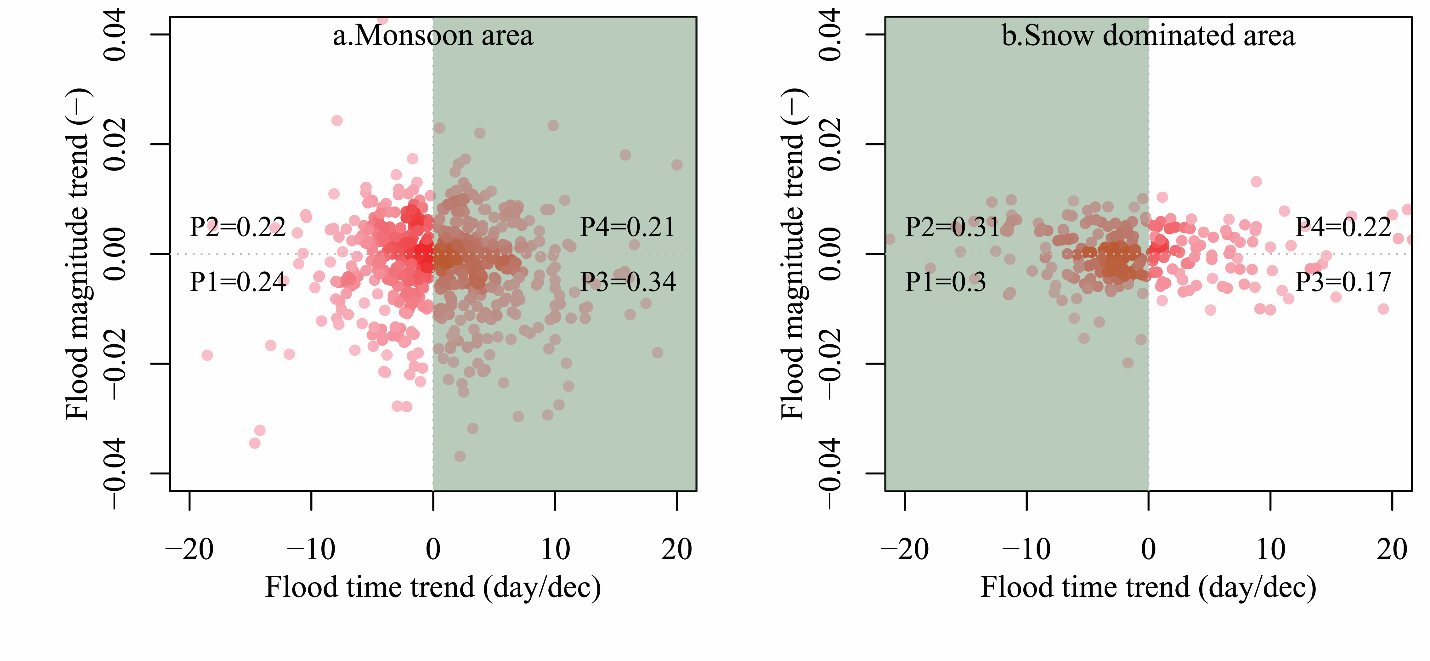


Figure S1 Relationships between flood timing change and flood magnitude change (in percent change of the mean annual flood discharge per decade) for the monsoon area and snowmelt affected area (defined as the ratio of the accumulated annual snowfall to annual runoff >0.5)[^17^](#_ENREF_17). The boundary of monsoon area is based on previous studies [^18^](#_ENREF_18). The flood magnitude changes were normalized by dividing the mean maximum flood of each station. Numbers labeled in each quadrant indicate the proportion of stations, e.g., P3=0.34 in Figure S1a represents that 34% stations showed delayed flood timing and decreased flood magnitude.

**References**

1. Do, H. X., Gudmundsson, L., Leonard, M. & Westra, S. The Global Streamflow Indices and Metadata Archive (GSIM) - Part 1: The production of a daily streamflow archive and metadata. *Earth System Science Data* **10**, doi:10.5194/essd-10-765-2018 (2018).

2. Gudmundsson, L., Do, H. X., Leonard, M. & Westra, S. The Global Streamflow Indices and Metadata Archive (GSIM) – Part 2: Quality control, time-series indices and homogeneity assessment. *Earth Syst. Sci. Data* **10**, 787-804, doi:10.5194/essd-10-787-2018 (2018).

3. Yatagai, A. *et al.* APHRODITE: Constructing a Long-Term Daily Gridded Precipitation Dataset for Asia Based on a Dense Network of Rain Gauges. *B Am Meteorol Soc* **93**, 1401-1415, doi:10.1175/BAMS-D-11-00122.1 (2012).

4. Harris, I. & Osborn, T. J. Version 4 of the CRU TS monthly high-resolution gridded multivariate climate dataset. **7**, 109, doi:10.1038/s41597-020-0453-3 (2020).

5. Beck, H. E. *et al.* Present and future Köppen-Geiger climate classification maps at 1-km resolution. *Scientific data* **5**, 180214, doi:10.1038/sdata.2018.214 (2018).

6. Singh, J., Ghosh, S., Simonovic, S. P. & Karmakar, S. Identification of flood seasonality and drivers across Canada. *Hydrol Process* **35**, doi:10.1002/hyp.14398 (2021).

7. Berghuijs, W., Woods, R., Hutton, C. & Sivapalan, M. Dominant flood generating mechanisms across the United States. *Geophys Res Lett* **43**, 4382-4390, doi:10.1002/2016GL068070 (2016).

8. Dethier, E. N., Sartain, S. L., Renshaw, C. E. & Magilligan, F. J. Spatially coherent regional changes in seasonal extreme streamflow events in the United States and Canada since 1950. *Science Advances* **6**, doi:10.1126/sciadv.aba5939 (2020).

9. Bartiko, D., Oliveira, D. Y., Bonumá, N. B. & Chaffe, P. L. B. Spatial and seasonal patterns of flood change across Brazil. *Hydrological Sciences Journal* **64**, 1071-1079, doi:10.1080/02626667.2019.1619081 (2019).

10. Cassalho, F., Beskow, S., de Mello, C. R., Oliveira, L. F. & de Aguiar, M. S. Evaluation of flood timing and regularity over hydrological regionalization in southern Brazil. *J Hydrol Eng* **24**, doi:10.1061/(asce)he.1943-5584.0001815 (2019).

11. Berghuijs, W. R., Harrigan, S., Molnar, P., Slater, L. J. & Kirchner, J. W. The relative importance of different flood-generating mechanisms across Europe. *Water Resour Res* **55**, 4582-4593, doi:https://doi.org/10.1029/2019WR024841 (2019).

12. Ficchi, A. & Stephens, L. Climate variability alters flood timing across Africa. *Geophys Res Lett* **46**, 8809-8819, doi:10.1029/2019gl081988 (2019).

13. Ganguli, P., Nandamuri, Y. R. & Chatterjee, C. Analysis of persistence in the flood timing and the role of catchment wetness on flood generation in a large river basin in India. *Theor Appl Climatol* **139**, 373-388, doi:10.1007/s00704-019-02964-z (2020).

14. Gu, X., Zhang, Q., Singh, V. P., Chen, X. & Liu, L. Nonstationarity in the occurrence rate of floods in the Tarim River basin, China, and related impacts of climate indices. *Global Planet Change* **142**, 1-13, doi:10.1016/j.gloplacha.2016.04.004 (2016).

15. Do, H. X., Westra, S., Leonard, M. & Gudmundsson, L. Global-scale prediction of flood timing using atmospheric reanalysis. *Water Resour Res* **56**, doi:10.1029/2019wr024945 (2020).

16. Wasko, C., Nathan, R. & Peel, M. C. Changes in Antecedent Soil Moisture Modulate Flood Seasonality in a Changing Climate. *Water Resour Res* **56**, doi:10.1029/2019wr026300 (2020).

17. Barnett, T. P., Adam, J. C. & Lettenmaier, D. P. Potential impacts of a warming climate on water availability in snow-dominated regions. *Nature* **438**, 303-309, doi:10.1038/nature04141 (2005).

18. Seneviratne, S. *et al.* Changes in climate extremes and their impacts on the natural physical environment. (Cambridge University Press, Cambridge, UK, and New York, NY, USA, 2012).
